# Cytotoxic Activity of Vancomycin-Resistant Enterococci Isolated from Hospitalised Patients

**DOI:** 10.3390/pathogens13100827

**Published:** 2024-09-25

**Authors:** Ewa Szczuka, Dominika Rolnicka, Maria Wesołowska

**Affiliations:** 1 Department of Microbiology, Faculty of Biology, Institute of Experimental Biology, Adam Mickiewicz University in Poznań, Uniwersytetu Poznańskiego 6, 61-614 Poznań, Poland; domrol@st.amu.edu.pl (D.R.); maria.wesolowska@amu.edu.pl (M.W.); 2 Microbiology Laboratory, University Clinical Hospital in Poznań, ul. Przybyszewskiego 49, 60-355 Poznań, Poland

**Keywords:** vancomycin-resistant enterococci (VRE), cytotoxic activity, drug resistance, AME genes

## Abstract

Vancomycin-resistant enterococci (VRE) are considered one of the main nosocomial pathogens due to their increasing antibiotic resistance and ability to cause life-threatening infections in humans. This study included VRE isolates obtained from various specimens including urine, blood, faeces, wounds, sputum, and oral cavity wash. Of the 37 strains, 30 (81.1%) and 7 (18.9%) were identified by MALDI TOF as *Enterococcus faecium* and *Enterococcus faecalis*, respectively. The clinical vancomycin-resistant enterococci exhibited multi-drug resistance (MDR). Apart from vancomycin, the enterococci exhibited resistance to penicillins (89.1 to 100%), fluoroquinolones (100%), rifampicin (86.5%), tetracycline (27%), aminoglycosides (56.8 to 86.5%), quinupristin–dalfopristin (35.1%), and chloramphenicol (10.8%). Moreover, resistance to linezolid and tigecycline emerged among the tested vancomycin-resistant enterococci. The analysis of aminoglycoside modifying enzyme (AME) genes showed the presence of bifunctional *aac(6*′*)-Ie-aph(2*″*)-Ia* genes contributed to high-level aminoglycoside resistance (HLAR) in the *E. faecalis* and *E. faecium* isolates. The other AME gene, i.e., *aph(3*′*)-IIIa*, was also found in the VRE isolates. All strains carried the *vanA* gene. Enterococci from colonised gastrointestinal tracts (1/2.7%) and from infection (6/16.2%) showed cytotoxic activity against the human epithelial cell line HEp-2.

## 1. Introduction 

Although enterococci are members of the human gastrointestinal microbiota, they may cause a variety of clinical infections, including bacteraemia, endocarditis, urinary tract infections, surgical wound infections, neonatal sepsis, and more rarely meningitis [1,2]. These infections most often occur in patients who have been hospitalised for a long period of time and immunocompromised patients. Enterococcal transmission occurs endogenously (translocation from the gut to the bloodstream) and exogenously, i.e., through direct contact with colonised or infected patients or health workers, or through contaminated equipment and environmental surfaces [3,4]. *E. faecalis* and *E. faecium* are implicated in the majority of healthcare-associated *Enterococcus* spp. infections [5,6]. The virulence factors that contribute to enterococcal pathogenesis include collagen-binding protein (Ace), aggregation substance (Agg), surface proteins (Esp), gelatinase (Gel), hyaluronidase, DNAse, and serine protease [3]. Furthermore, highly virulent enterococci synthesise cytolysin, which can damage host cell membranes and facilitate infection. The presence of cytolysin also promotes the appearance of enterococci in the bloodstream [7]. Patients with bacteraemia caused by toxin-harbouring *E. faecalis* strains had a five-fold increase in the risk of death within 3 weeks of infections compared to patients infected by non-cytolytic strains [8]. The enhanced virulence of *E. faecalis* due to cytolysin expression has been documented in mouse models and in a rabbit endocarditis model [7]. Xiong et al. [9] showed that a toxin-harbouring strain of *E. faecium* induced the death of peripheral blood mononuclear cells and damaged intestinal organoids, and this toxicity was neutralised by an antibody-toxin, which indicates the toxin-mediated virulence of E. *faecium*. The cytolysin operon is found on self-transmissible plasmids and within pathogenicity islands (PAI) on the chromosome of *E. faecalis* strains [10]. In addition to cytolysin, *E. faecalis* and *E. faecium* have also been shown to produce toxic oxygen metabolites that can cause cell damage [10]. In particular, *E. faecalis* strains produce superoxide (O_2_^−^), and their effects may lead to cellular damage in intestinal epithelial cells [3].

The emergence of VRE has limited therapeutic options and has become an important public health concern. Nine different vancomycin resistance gene clusters (*vanA*, *-B*, *-C*, *-D*, *-E*, *-G*, *-L*, *-M*, and *N*) have been identified. However, the *vanA* and *vanB* genotypes are the main vancomycin resistance genotypes in human infections [11]. The mechanism of glycopeptide resistance involves the alteration of peptidoglycan synthesis, i.e., the substitution of D-Alanine-D-Alanine (D-Ala-D-Ala) with D-Alanine-D-Lactate (D-Ala-D-Lac) in the *vanA* and *vanB* genotype. Vancomycin binds to D-Ala-D-Lac with a 1000-fold lower affinity than D-Ala-D-Ala, which mediates high resistance [12]. Aminoglycosides are traditionally used in combination with β-lactams to treat enterococcal infections. It is known that a cell-wall-active antibiotic that inhibits the synthesis of peptidoglycan increases cell wall permeability and increases the uptake of aminoglycosides. Unfortunately, high-level aminoglycoside resistance (HLAR), mediated by aminoglycoside modifying enzymes (AMEs), particularly a bifunctional enzyme encoded by *aac(6*′*)-Ie-aph(2*″*)-Ia*, has spread through clinical isolates. Enterococci that encode the AAC(6′)-APH(2″) enzyme are resistant to all of the available clinical aminoglycosides, including gentamicin, tobramycin, amikacin, and kanamycin, except streptomycin [13]. The ANT(6′)-Ia enzyme appears to be most commonly associated with high-level streptomycin resistance. High resistance to streptomycin was also associated with punctual ribosome mutations [14]. Therapeutic alternatives for the VRE infections are restricted to daptomycin, tigecycline, and linezolid. However, it is important to note that the value of daptomycin has been found to be limited by the rate of development of daptomycin resistance during VRE treatment [15,16].

The main objective of this study was to determine the cytotoxic activity of VRE isolates on human epithelial cells, HEp-2. In addition, we analysed the antibiotic susceptibility of enterococci, identified the *van* genotype, and examined the presence of genes encoding AMEs.

## 2. Material and Methods

### 2.1. Bacterial Strains

A total of 37 *Enterococcus* strains were isolated from specimens collected from patients of the University Clinical Hospital in Poznań from January to December 2022. Most isolated bacterial strains were obtained from faeces (*n* = 13; 35.1%), followed by urine (*n* = 12; 32.4%), blood (*n* = 5; 13.5%), wounds (*n* = 4; 10.8%), the oral cavity (*n* = 1; 2.7%), and sputum (*n* = 2; 5.4%) (Table S1). The strains from faeces, the oral cavity, and sputum were regarded as non-invasive ones (without symptoms of infection) (*n* = 16; 43.2%). The isolates from urine, blood, and wounds were responsible for symptomatic infections, and they were found to be invasive (*n* = 21, 56.8%). The isolates were identified with conventional methods (Gram stain and catalase test) and MALDI-TOF MS (matrix-assisted laser desorption ionisation time-of-flight mass spectrometry) (Bruker, Billerica, MA, USA).

### 2.2. Susceptibility Testing

Susceptibility testing was performed using the standard disc agar diffusion method in accordance with the European Committee on Antimicrobial Susceptibility Testing (EUCAST) guidelines. A bacterial culture (100 µL) was streaked onto Mueller–Hinton agar (Oxoid) plates using swabs and incubated at 37 °C for 18–24 h. The antimicrobial agents (Biomereux) used in this study were penicillin (10 units), ampicillin (2 μg), vancomycin (5 μg), teicoplanin (30 μg), gentamicin (30 μg), streptomycin (300 μg), tigecycline (15 μg), doxycycline (30 μg), ciprofloxacin (5 μg), levofloxacin (5 μg), chloramphenicol (30 μg), rifampicin (5 μg), linezolid (10 μg), and quinupristin–dalfopristin (15 μg). The inhibitory zones were measured after the incubation, and the findings were classed as sensitive or resistant using the EUCAST or CLSI (when EUCAST breakpoints were not available) interpretation criteria [17,18]. *E. faecalis* ATCC 29212 was used as a control strain in the disc agar diffusion test.

### 2.3. Preparation of Total DNA for PCR and Detection of Antibiotic-Resistant Genes

The total DNA was isolated and purified using the Genomic Mini DNA kit (A&A Biotechnology, Gdynia, Poland). The presence of *vanA* (primer sequences: F: CAT GAA TAG AAT AAA AGT TGC AAT A and R: CCC CTT TAA CGC TAA TAC GAT CAA), *vanB* (primer sequences: F: GTG ACA AAC CGG AGG CGA GGA and R: CCG CCA TCC TCC TGC AAA AAA)*, vanC* (primer sequences: F: GAA AGA CAA CAG GAA GAC CGC and R: ATC GCA TCA CAA GCA CCA ATC), and AME genes was assessed using a PCR assay as previously described [19,20].

### 2.4. Cytotoxic Activity to Human Epithelial Cells (HEp-2)

The MTT assay was used to assess the cytotoxic effect of the cytotoxin-producing strains on the HEp-2 cell line [21]. Briefly, overnight cultures in TSB were centrifuged and the supernatants were sterilised through a membrane filter with a pore size of 0.22 μm. A monolayer of HEp-2 cells was incubated with 100μL of supernatant, for 24 h at 37 °C. Cytotoxicity was measured by means of the mitochondrial-dependent reduction of colourless 3-(4,5-dimethylthiazol-2-yl)-2,5-diphenyltetrazolium bromide (MTT; Sigma, St. Louis, MO, USA) to blue formazan, which was dissolved in a mixture of isopropanol and HCl. Absorbance at λ = 550 nm was measured with a spectrophotometer. Cells treated with non-pathogenic *E. coli* K12C600 filtrate were used as a negative control.

## 3. Results

### 3.1. Species Identification and Van Genotype Determinations

Of the 37 isolates, 30 (81.1%) and 7 (18.9%) were identified by MALDI TOF as *Enterococcus faecium* and *Enterococcus faecalis,* respectively. The enterococcal isolates harboured the *vanA* gene, and none of them harboured the *vanB* and *vanC* genes. All strains were resistant to vancomycin and teicoplanin. Nearly half of these isolates (43.2%) were obtained from the colonised patients, while 56.8% of the strains were responsible for bacteraemia, urinary tract infections, and skin infections (Table 1).

### 3.2. Antimicrobial Susceptibility Testing

The vancomycin-resistant enterococci exhibited multi-drug resistance (MDR). Their resistance profiles are shown in Table 1. The highest frequency of resistance was observed for penicillin (100%) and fluoroquinolones, i.e., ciprofloxacin (100%) and levofloxacin (100%) (Tables S2 and S3). More than 85% of the isolates showed resistance to ampicillin. High-level resistance to gentamicin was detected in 56.8% of the strains, and to streptomycin in 86.5% of the strains. The *aac(6*′*)-Ie-aph(2″)-Ia* gene, which mediates high-level gentamicin resistance, was found in 21 isolates, while *aph(3*′*)-IIIa* genes were found in 17 isolates. In the analysed enterococci, we did not find the *aph(2″)-Ib, aph(2″)-Ic,* and *aph(2″)-Id* genes, which encode other aminoglycoside-modifying enzymes and are also known to mediate enterococcal resistance to gentamicin. We also did not find *ant(4*′*)-Ia* genes, which encode resistance of enterococci to various aminoglycosides but not to gentamicin and streptomycin. The data obtained in this study revealed that the VRE isolates exhibited resistance to rifampicin (86.5%), quinupristin–dalfopristin (35.1%), doxycycline (27%), and chloramphenicol (10.8%). Importantly, one strain of *E. faecium* (MPU E7) isolated from wounds showed resistance to linezolid. One tigecycline-resistant strain of *E. faecalis* (MPU E14) was also reported in this study.

### 3.3. Cytotoxic Activity

Seven VRE isolates (18.9%) had cytotoxic effects on the HEp-2 cells, and between 10 and 62% of the cells were destroyed (Table 1). The highest activity (>60% of cells destroyed) was observed for two strains responsible for UTIs, i.e., *E. faecalis* (MPU E2) and *E. faecium* (MPU E1). The lowest cytotoxicity (less than 20% of cells destroyed) was observed for two *E. faecium* MPU E5 and MPU E6 strains isolated from urine and blood. Another *E. faecium* strain (MPU E34) isolated from blood and one strain isolated from wounds (MPU E7) damaged 26% and 46% of the HEp-2 cells, respectively. Of note, only one strain (MPU E24) originating from colonisation damaged the human epithelial cells.

## 4. Discussion

Haemato-oncological patients undergoing long-term hospitalisation constitute a significant group of patients from whom VRE strains were isolated in our study. Recently, Zerbato et al. [22] reported that the 30-day mortality rate was higher for vancomycin-resistant *E. faecium* nosocomial bloodstream infections (BSIs) than for vancomycin-sensitive *E. faecium* BSIs. Enterococci from colonised gastrointestinal and oral mucous membranes can be responsible for endogenous infections such as UTIs and bacteraemia. In Italy, studies have shown that the oral cavity can be regarded as a reservoir of clinically relevant enterococci. However, healthy individuals rarely carry vancomycin-resistant strains [23]. In our study, vancomycin-resistant *E. faecium* was isolated more frequently than *E. faecalis*. Our findings are in agreement with previous studies demonstrating a higher incidence of vancomycin-resistant *E. faecium* among haematology and oncology patients [6,24,25]. Similar to other European countries, the *vanA* phenotype dominated among the enterococcal strains [2,16,24,25]. Importantly, the *vanA* gene cluster is frequently present in plasmids, which increases the risk of dissemination of vancomycin resistance among clinical strains [11].

The VRE strains included in this study exhibited resistance to a variety of antimicrobial agents. It appears that almost all the isolates showed resistance to penicillins (i.e., ampicillin), which generally have the greatest activity against enterococci. Several previous studies indicate a high prevalence of resistance to ampicillin in European countries [5]. For example, in Greece, the resistance to ampicillin increased from 92 to 100% during five years of study [26]. Interestingly, the high rate (88%) of resistance to ampicillin was also reported among *S. faecium* strains isolated from hospitalised patients on the Pacific Islands [27]. In the current study, all the *E. faecium and E. faecalis* strains exhibited resistance to quinolones (i.e., ciprofloxacin and levofloxacin). Recently, Gawryszewska et al. [28] have reported a lack of sensitivity to ciprofloxacin among linezolid-resistant enterococci collected over a period of 7 years in Poland. We found that over half of the isolates showed high-level resistance to gentamycin, and the majority exhibit high-level resistance to streptomycin. Similar to our results, about half of *Enterococcus* spp. isolates from urinary tract infections showed resistance to gentamicin [29]. In contrast, HLR to gentamicin was detected in 10.5% of *E. faecium* strains isolated from human invasive infections during a 5-year period in Argentina [30]. The genes conferring aminoglycoside resistance were detected in 64.8% of the enterococcal strains. The most common AME gene was *aac(6*′*)-Ie-aph(2″)-Ia,* coding for the bifunctional enzyme AAC(6′)-APH(2″), which confers resistance to all available aminoglycosides, except streptomycin. This is in agreement with previous data from China showing that *aac(6*′*)-Ie-aph(2″)-Ia* is the most prevalent gene among *Enterococcus* spp. [31]. Another AME gene, i.e., *aph(3*′*)-IIIa*, which confers resistance to amikacin and kanamycin, was identified in the VRE isolates. The current study has shown that *E. faecium* and *E. faecalis* strains isolated from hospitalised patients are resistant to important anti-enterococcal drugs, such as penicillin, aminoglycosides, and glycopeptides. Therefore, infections caused by these strains are difficult to treat. As mentioned above, the alternative is linezolid, which is effective in the treatment of VRE bacteraemia and endocarditis [32]. In this study, one isolate of *E. faecium* showed resistance to linezolid. It should be noted that linezolid-resistant enterococci to have been reported in many countries of Europe, Asia, and Central and South America. Fortunately, the resistance to linezolid remains relatively low [32,33]. Another therapeutic option for treating VRE infections, especially skin and soft-tissue infections, is tigecycline. However, the use of tigecycline as a monotherapy is discouraged due to the emergence of resistance during therapy [16]. Tigecycline resistance was identified in the current study, in only one *E. faecalis* isolate. Other studies have occasionally reported the emergence of tigecycline resistance in *E. faecium* and *E. faecalis* [32].

Another interesting finding of our study is the cytotoxic activity of 18.9% of the VRE isolates. Cytolysis is of interests because it enhances virulence through the lysis of erythrocytes or destruction of other host cells. To the best of our knowledge, this is the first study showing the cytotoxic activity of VRE strains on the human epithelial cell line, HEp-2. Our results indicated that five *E. faecium* and two *E. faecalis* strains showed cytotoxic activity. Previous studies reported the presence of a cytolysis operon in the genomes of 5 out of 17 (29%) endophthalmitis isolates and the ability of these strains to induce haemolysis when grown in the presence of horse blood [34]. Another study indicated that the gene *cylA* coding for cytolysin was present in 62% of *E. faecalis* and in 56% of *E. faecium* strains collected from hospitalised patients [35]. This study revealed that only one strain from intestinal colonisation had cytotoxic activity. Interestingly, 38% of *Enterococcus* spp. strains isolated from oral cavity colonisation showed haemolytic activity on Mueller–Hinton agar supplemented with 5% human blood [36].

Our work has some limitations that must be taken into account when interpreting the results. Firstly, only VRE strains were included in this study. Secondly, only a low number of *E. faecalis* were analysed. During this period of study, *E. faecium* appears to be the most common among VRE. In the future, we will also focus on other *Enterococcus* species than *E. faecalis* or *E. faecium*. In this study, intestinal colonisation with vancomycin-resistant *E. faecium* and *E. faecalis* was demonstrated. In addition, strains from colonised patients as well as from patients with bacteraemia, urinary tract infections, and skin infections showed multi-drug resistance. The therapeutic options for the treatment of VRE infections are linezolid and tigecycline. However, linezolid resistance and tigecycline resistance emerged among the tested enterococci.

## 5. Conclusions

The clinical VRE strains exhibited multi-drug resistance, with high-level resistance to aminoglycosides. The enterococci harboured AME genes, i.e., the *aac(6*′*)-Ie-aph(2″)-Ia* and *aph(3*′*)-IIIa* genes. In addition, linezolid resistance and tigecycline resistance emerged among the tested vancomycin-resistant enterococci. This study revealed that *E. faecium* and *E. faecalis* showed cytotoxic activity against human epithelial cells, HEp-2.

## Figures and Tables

**Table 1:** Antimicrobial resistance profiles, distribution of antibiotic resistance genes, and cytotoxic activity against HEp-2 of VRE isolates.

Isolate ID	Species	Origin	Antimicrobial Resistance Profile	*Van* Genotype	Prevalence of AME Genes						Cytotoxic Activity
					*aac(6*′*)-Ie-aph(2″)-Ia*	*aph(2″)* *-Ib*	*aph(2″)-Ic*	*aph(2″)-Id*	*aph(3*′*)-IIIa*	*ant(4*′*)-Ia*	
MPU E 1	*E. faecium*	Urine	VAN, TEC, P, AMP, GN, S, CIP, LEV, RD	*vanA*	+	−	−	−	+	−	+
MPU E 2	*E. faecalis*	Urine	VAN, TEC, P, AMP, S, CIP, LEV, RD, QD	*vanA*	−	−	−	−	+	−	+
MPU E 3	*E. faecium*	Urine	VAN, TEC, P, AMP, GN, CIP, LEV, RD	*vanA*	+	−	−	−	−	−	−
MPU E 4	*E. faecium*	Faeces	VAN, TEC, P, AMP, GN, S, CIP, LEV, RD, QD	*vanA*	+	−	−	−	+	−	−
MPU E 5	*E. faecium*	Blood	VAN, TEC, P, AMP, S, DOX, CIP, LEV, RD	*vanA*	−	−	−	−	−	−	+
MPU E 6	*E. faecium*	Urine	VAN, TEC, P, AMP, S, CIP, LEV, RD, QD	*vanA*	−	−	−	−	−	−	+
MPU E 7	*E. faecium*	Wounds	VAN, TEC, P, AMP, GN, S, CIP, LEV, C, RD, QD, LZD	*vanA*	+	−	−	−	−	−	+
MPU E 8	*E. faecium*	Urine	VAN, TEC, P, AMP, GN, S, DOX, CIP, LEV, C, RD, QD	*vanA*	+	−	−	−	+	−	−
MPU E 9	*E. faecalis*	Blood	VAN, TEC, P, GN, S, CIP, LEV, QD	*vanA*	+	−	−	−	+	−	−
MPU E 10	*E. faecium*	Urine	VAN, TEC, P, AMP, GN, S, CIP, LEV, RD	*vanA*	+	−	−	−	+	−	−
MPU E 11	*E. faecium*	Urine	VAN, TEC, P, AMP, GN, S, CIP, LEV, RD, QD	*vanA*	+	−	−	−	+	−	−
MPU E 12	*E. faecium*	Wounds	VAN, TEC, P, AMP, S, DOX, CIP, LEV, RD	*vanA*	−	−	−	−	−	−	−
MPU E 13	*E. faecalis*	Urine	VAN, TEC, P, GN, CIP, LEV, QD	*vanA*	+	−	−	−	−	−	−
MPU E 14	*E. faecalis*	Blood	VAN, TEC, P, GN, S, CIP, LEV, QD, TIG	*vanA*	+	−	−	−	+	−	−
MPU E 15	*E. faecium*	Urine	VAN, TEC, P, AMP, GN, S, DOX, CIP, LEV, RD	*vanA*	+	−	−	−	−	−	−
MPU E 16	*E. faecium*	Sputum	VAN, TEC, P, AMP, GN, S, CIP, LEV, RD	*vanA*	+	−	−	−	−	−	−
MPU E 17	*E. faecium*	Urine	VAN, TEC, P, AMP, CIP, LEV, RD, QD	*vanA*	−	−	−	−	−	−	−
MPU E 18	*E. faecium*	Faeces	VAN, TEC, P, AMP, GN, S, DOX, CIP, LEV, RD	*vanA*	+	−	−	−	+	−	−
MPU E 19	*E. faecium*	Urine	VAN, TEC, P, AMP, S, CIP, LEV, RD	*vanA*	−	−	−	−	+	−	−
MPU E 20	*E. faecium*	Faeces	VAN, TEC, P, AMP, GN, S, DOX, CIP, LEV, RD	*vanA*	+	−	−	−	+	−	−
MPU E 21	*E. faecium*	Faeces	VAN, TEC, P, AMP, GN, S, CIP, LEV, RD	*vanA*	+	−	−	−	+	−	−
MPU E 22	*E. faecium*	Urine	VAN, TEC, P, AMP, S, CIP, LEV, RD	*vanA*	−	−	−	−	−	−	−
MPU E 23	*E. faecalis*	Faeces	VAN, TEC, P, AMP, GN, S, CIP, LEV, RD, QD	*vanA*	+	−	−	−	+	−	−
MPU E 24	*E. faecalis*	Faeces	VAN, TEC, P, GN, S, CIP, LEV, QD	*vanA*	+	−	−	−	−	−	+
MPU E 25	*E. faecalis*	Sputum	VAN, TEC, P, AMP, CIP, LEV, RD	*vanA*	−	−	−	−	−	−	−
MPU E 26	*E. faecium*	Faeces	VAN, TEC, P, AMP, S, DOX, CIP, LEV, RD	*vanA*	−	−	−	−	−	−	−
MPU E 27	*E. faecium*	Faeces	VAN, TEC, P, AMP, S, DOX, CIP, LEV, RD	*vanA*	−	−	−	−	−	−	−
MPU E 28	*E. faecium*	Faeces	VAN, TEC, P, AMP, S, CIP, LEV, C	*vanA*	−	−	−	−	−	−	−
MPU E 29	*E. faecium*	Faeces	VAN, TEC, P, AMP, GN, S, CIP, LEV, C, RD	*vanA*	+	−	−	−	+	−	−
MPU E 30	*E. faecium*	Blood	VAN, TEC, P, AMP, S, CIP, LEV, RD	*vanA*	−	−	−	−	−	−	−
MPU E 31	*E. faecium*	Wounds	VAN, TEC, P, AMP, GN, S, CIP, LEV, RD	*vanA*	+	−	−	−	+	−	−
MPU E 32	*E. faecium*	Wounds	VAN, TEC, P, AMP, S, CIP, LEV, RD	*vanA*	−	−	−	−	−	−	−
MPU E 33	*E. faecium*	Oral cavity	VAN, TEC, P, AMP, S, CIP, LEV, RD	*vanA*	−	−	−	−	−	−	−
MPU E 34	*E. faecium*	Blood	VAN, TEC, P, AMP, S, DOX, CIP, LEV, RD	*vanA*	−	−	−	−	−	−	+
MPU E 35	*E. faecium*	Faeces	VAN, TEC, P, AMP, CIP, DOX, LEV, QD, RD	*vanA*	−	−	−	−	−	−	−
MPU E 36	*E. faecium*	Faeces	VAN, TEC, P, AMP, GN, S, CIP, LEV, RD	*vanA*	+	−	−	−	+	−	−
MPU E 37	*E. faecium*	Faeces	VAN, TEC, P, AMP, GN, S, CIP, LEV RD	*vanA*	+	−	−	−	+	−	−

Van, vancomycin; TEC, teicoplanin; P, penicillin; AMP, ampicillin; GN, gentamicin; S, streptomycin; DOX, doxycycline; CIP, ciprofloxacin; LEV, levofloxacin; C, chloramphenicol; RD, rifampicin; QD, quinupristin–dalfopristin; TIG, tigecycline; LZD, linezolid.

## Data Availability

The original contributions presented in the study are included in the article/Supplementary Material, further inquiries can be directed to the corresponding author.

## Supplementary Materials

The following supporting information can be downloaded at: https://www.mdpi.com/article/10.3390/pathogens13100827/s1, Table S1: The origin of *E. faecium* and *E. faecalis* strains. Table S2: Antimicrobial resistance of *E. faecium* isolated from hospitalised patients. Table S3: Antimicrobial resistance of *E. faecalis* isolated from hospitalised patients.

## References

[1] Ramos S., Silva V., Dapkevicius M., Igrejas G., Poeta P. (2020). Enterococci, from harmless bacteria to a pathogen. Microorganisms.

[2] Dubin K., Pamer E.G. (2016). Enterococci and their interactions with the intestinal microbiome. Microbiol. Spectr..

[3] Fiore E., Van Tyne D., Gilmore M.S. (2019). Pathogenicity of enterococci. Microbiol. Spectr..

[4] Pandova M., Kizheva Y., Tsenova M., Rusinova M., Borisova T., Hristova P. (2024). Pathogenic potential and antibiotic susceptibility: A comprehensive study of Enterococci from different ecological settings. Pathogens.

[5] Zaheer R., Cook S.R., Barbieri R., Goji N., Cameron A., Petkau A., Polo R.O., Tymensen L., Stamm C., Song J. (2020). Surveillance of *Enterococcus* spp. reveals distinct species and antimicrobial resistance diversity across a One-Health continuum. Sci. Rep..

[6] Top J., Willems R., Bonten M. (2008). Emergence of CC17 *Enterococcus faecium*: From commensal to hospital-adapted pathogen. FEMS Immunol. Med. Microbiol..

[7] Van Tyne D., Martin M.J., Gilmore M.S. (2013). Structure, function, and biology of the *Enterococcus faecalis* cytolysin. Toxins.

[8] Huycke M.M., Spiegel C.A., Gilmore M.S. (1991). Bacteremia caused by hemolytic, high-level gentamicin-resistant enterococcus faecalis. Antimicrob. Agents Chemother..

[9] Xiong X., Tian S., Yang P., Lebreton F., Bao H., Sheng K., Yin L., Chen P. (2022). Emerging enterococcus pore-forming toxins with MHC/HLA-I as receptors. Cell.

[10] Coburn P.S., Gilmore M.S. (2003). The *Enterococcus faecalis* cytolysin: A novel toxin active against eukaryotic and prokaryotic cells. Cell Microbiol..

[11] Ahmed M.O., Baptiste K.E. (2017). Vancomycin-resistant enterococci: A review of antimicrobial resistance mechanisms and perspectives of human and animal health. Microb. Drug Resist..

[12] Miller W.R., Munita J.M., Arias C.A. (2014). Mechanisms of antibiotic resistance in enterococci. Expert Rev. Anti. Infect. Ther..

[13] Chow J.W. (2001). Aminoglycoside resistance in enterococci. Clin. Infect. Dis..

[14] Li G., Walker M.J., De Oliveira D.M.P. (2023). Vancomycin resistance in *Enterococcus* and *Staphylococcus aureus*. Microorganisms.

[15] Geraldes C., Tavares L., Gil S., Oliveira M. (2022). *Enterococcus* virulence and resistant traits associated with its permanence in the hospital environment. Antibiotics.

[16] Herrera-Hidalgo L.H., Fernández-Rubio F., Luque-Márquez R., López-Cortés L.E., Gil-Navarro M.V., de Alarcón A. (2023). Treatment of *Enterococcus faecalis* infective endocarditis: A continuing challenge. Antibiotics.

[17] EUCAST Version 13.1, 2023. (n.d.). http://www.eucast.org/expert_rules_and_intrinsic_resistance.

[18] (2022). Performance Standards for Antimicrobial Susceptibility Testing, 32nd ed.

[19] Clark N.C., Cooksey R.C., Hill B.C., Swenson J.M., Tenover F.C. (1993). Characterization of glycopeptide-resistant enterococci from U.S. hospitals. Antimicrob. Agents Chemother..

[20] Vakulenko S.B., Donabedian S.M., Voskresenskiy A.M., Zervos M.J., Lerner S.A., Chow J.W. (2003). Multiplex PCR for detection of aminoglycoside resistance genes in enterococci. Antimicrob. Agents Chemother..

[21] Szczuka E., Jabłońska L., Kaznowski A. (2016). Coagulase-negative staphylococci: Pathogenesis, occurrence of antibiotic resistance genes and in vitro effects of antimicrobial agents on biofilm-growing bacteria. J. Med. Microbiol..

[22] Zerbato V., Pol R., Sanson G., Suru D.A., Pin E., Tabolli V., Monticelli J., Busetti M., Toc D.A., Crocè L.S. (2024). Risk factors for 30-day mortality in nosocomial enterococcal bloodstream infections. Antibiotics.

[23] Palmieri A., Martinelli M., Pellati A., Carinci F., Lauritano D., Arcuri C., Baggi L., Gatto R., Luca Scapoli L. (2023). Prevalence of Enterococci and vancomycin resistance in the throat of non-hospitalized individuals randomly selected in central Italy. Antibiotics.

[24] Pinholt M., Gumpert H., Bayliss S., Nielsen J.B., Vorobieva V., Pedersen M., Feil E., Worning P., Westh H. (2017). Genomic analysis of 495 vancomycin-resistant *Enterococcus faecium* reveals broad dissemination of a *vanA* plasmid in more than 19 clones from Copenhagen, Denmark. J. Antimicrob. Chemother..

[25] Krawczyk B., Wysocka M., Kotłowski R., Marek Bronk M., Michalik M., Samet A. (2020). Linezolid-resistant *Enterococcus faecium* strains isolated from one hospital in Poland -commensals or hospital-adapted pathogens?. PLoS ONE.

[26] Tsalidou M., Stergiopoulou T., Bostanitis I., Nikaki C., Skoumpa K., Koutsoukou T., Papaioannidou P. (2023). Surveillance of antimicrobial resistance and multidrug resistance prevalence of clinical isolates in a regional hospital in Northern Greece. Antibiotics.

[27] Strobel A.G., Prasad P., Prasad V., Ravi Naidu R., Young-Sharma T., Ana Suka A., Richards M., Cameron D., Lane C.R., Buising K. (2024). The epidemiology of enterococci in a tertiary hospital and primary healthcare facilities in Fiji (2019–2022). J. Glob. Antimicrob. Resist..

[28] Gawryszewska I., Żabińska D., Hryniewicz W., Sadowy E. (2017). Linezolid-resistant enterococci in Polish hospitals: Species, clonality and determinants of linezolid resistance. Eur. J. Clin. Microbiol. Infect. Dis..

[29] Khalil M.A., Alorabi J.A., Al-Otaibi L.M., Ali S.S., Elsilk S.E. (2023). Antibiotic resistance and biofilm formation in *Enterococcus* spp. isolated from urinary tract infections. Pathogens.

[30] Schell C.M., Tedim A.P., Rodríguez-Baños M., Sparo M.D., Lissarrague S., Basualdo J.A., Teresa M., Coque T.M. (2020). Detection of β-lactamase-producing *Enterococcus faecalis* and vancomycin-resistant *Enterococcus faecium* isolates in human invasive infections in the Public Hospital of Tandil, Argentina. Pathogens.

[31] Tian Y., Yu H., Wang Z. (2019). Distribution of acquired antibiotic resistance genes among *Enterococcus* spp. isolated from a hospital in Baotou, China. BMC Res. Notes.

[32] Dadashi M., Sharifian P., Bostanshirin N., Hajikhani B., Bostanghadiri N., Khosravi-Dehaghi N., van Belkum A., Darban-Sarokhalil D. (2021). The global prevalence of daptomycin, tigecycline, and linezolid-Resistant *Enterococcus faecalis* and *Enterococcus faecium* Strains from Human Clinical Samples: A Systematic Review and Meta-Analysis. Front. Med..

[33] Mendes R.E., Deshpande L., Streit J.M., Sader H.S., Castanheira M., Hogan P.M., Flamm R.K. (2018). ZAAPS programme results for 2016: An activity and spectrum analysis of linezolid using clinical isolates from medical centres in 42 countries. J. Antimicrob. Chemother..

[34] Chilambi G.S., Nordstrom H.R., Evans D.R., Kowalski R.P., Dhaliwal D.K., Vishal Jhanji V., Shanks R.M.Q., Van Tyne D. (2021). Genomic and phenotypic diversity of *Enterococcus faecalis* isolated from endophthalmitis. PLoS ONE.

[35] Backiam A.D.S., Duraisamy S., Karuppaiya P., Balakrishnan S., Chandrasekaran B., Kumarasamy A., Raju A. (2023). Antibiotic susceptibility patterns and virulence-associated factors of vancomycin-resistant enterococcal isolates from Tertiary Care Hospitals. Antibiotics.

[36] Komiyama E.Y., Lepesqueur L.S.S., Yassuda C.G., Samaranayake L.P., Parahitiyawa N.B., Balducci I., Koga-Ito C.Y. (2016). *Enterococcus* species in the oral cavity: Prevalence, virulence factors and antimicrobial susceptibility. PLoS ONE.

