# Supplementary material for: Cytotoxic Activity of Vancomycin-Resistant Enterococci Isolated from Hospitalised Patients
Source: Pathogens. 2024 Sep 25;13(10):827. doi: 10.3390/pathogens13100827 (PMC11509928; doi:10.3390/pathogens13100827)
Supplement: Supplementary file 1 [file pathogens-13-00827-s001.zip › pathogens-3119450-supplementary.pdf]

Table S1. The origin of *E. faecium* and *E. faecalis* strains.

Origin	Number of *E. faecium* strains (%)	Number of *E. faecalis* strains (%)
faeces	11 (36.7)	2 (28.6)
urine	10 (33.3)	2 (28.6)
blood	3 (10)	2 (28.6)
wounds	4 (13.3)	0
oral cavity	1 (3.3)	0
sputum	1 (3.3)	1 (14.2)

Table S2. Antimicrobial resistance of *E. faecium* isolated from hospitalised patients.

Antibiotic class	Antimicrobial drug	Antimicrobial susceptibility					
		Resistant		Intermediate		Sensitive	
		n	%	n	%	n	%
Glycopeptide	Vancomycin	30	100.00	0	0.00	0	0.00
	Teicoplanin	30	100.00	0	0.00	0	0.00
β-lactams	Penicillin	30	100.00	0	0.00	0	0.00
	Ampicillin	30	100.00	0	0.00	0	0.00
Aminoglycosides	Gentamycin	16	53.33	0	0.00	14	46.67
	Streptomycin	27	90.00	0	0.00	3	10.00
Tetracyclines	Doxycycline	10	33.33	0	0.00	20	66.67
Fluoroquinolones	Ciprofloxacin	30	100.00	0	0.00	0	0.00
	Levofloxacin	30	100.00	0	0.00	0	0.00
Chloramphenicol	Chloramphenicol	4	13.33	0	0.00	26	86.67
Rifampicin	Rifampicin	29	96.67	0	0.00	1	3.33
Streptogramins	Quinupristin-dalfopristin	8	26.67	0	0,00	22	73.33
Glycylcyclines	Tigecycline	0	0.00	0	0.00	30	100.00
Oxazolidinones	Linezolid	1	3.33	0	0.00	29	96.67

Table S3. Antimicrobial resistance of *E. faecalis* isolated from hospitalised patients.

Antibiotic class	Antimicrobial drug	Antimicrobial susceptibility					
		Resistant		Intermediate		Sensitive	
		n	%	n	%	n	%
Glycopeptides	Vancomycin	7	100.00	0	0.00	0	0.00
	Teicoplanin	7	100.00	0	0.00	0	0.00
β-lactams	Penicillin	7	100.00	0	0.00	0	0.00
	Ampicillin	3	42.86	0	0.00	4	57.14
Aminoglycosides	Gentamycin	5	71.43	0	0.00	2	28.57
	Streptomycin	5	71.43	0	0.00	2	28.57
Tetracyclines	Doxycycline	0	0	0	0.00	7	100.00
Fluoroquinolones	Ciprofloxacin	7	100.00	0	0.00	0	0.00
	Levofloxacin	7	100.00	0	0.00	0	0.00
Chloramphenicol	Chloramphenicol	0	0.00	0	0.00	7	100.00
Rifampicin	Rifampicin	3	42.86	0	0.00	4	57.14
Streptogramins	Quinupristin-dalfopristin	5	71.43	0	0.00	2	28.57
Glycylcyclines	Tigecycline	1	14.29	0	0.00	6	85.71
Oxazolidinones	Linezolid	0	0.00	0	0.00	7	100.00
